# Diet quality, consumption of seafood and eggs are associated with sleep quality among Chinese urban adults: A cross‐sectional study in eight cities of China

**DOI:** 10.1002/fsn3.1050

**Published:** 2019-05-15

**Authors:** Wei Wu, Ai Zhao, Ignatius Man‐Yau Szeto, Yan Wang, Liping Meng, Ting Li, Jian Zhang, Meichen Wang, Zixing Tian, Yumei Zhang

**Affiliations:** ^1^ Department of Nutrition and Food Hygiene, School of Public Health Peking University Health Science Center Beijing China; ^2^ Department of Social Science and Health Education, School of Public Health Peking University Health Science Center Beijing China; ^3^ Inner Mongolia Dairy Technology Research Institute Co. Ltd Hohhot China; ^4^ Yili Innovation Center Inner Mongolia Yili Industrial Group Co. Ltd Hohhot China

**Keywords:** diet quality, eggs, food, seafood, sleep quality

## Abstract

Growing evidence has suggested that dietary modification is implicated with sleep alteration. Our study aimed to determine whether an association between diet in terms of diet quality, certain food consumption, and dietary nutrients intake and sleep quality existed in Chinese urban adults, which has been fully investigated. A cross‐sectional study was conducted among urban adults from eight Chinese cities. Total of 1,548 participants remained in the final analysis. Sleep quality was evaluated by the Chinese version of the Pittsburg Sleep Questionnaire Index. Diet quality, evaluated by Chinese Healthy Diet Index, and dietary intake, including food groups and nutrients, were derived from a semiquantitative Food Intake Frequencies Questionnaire and a single 24‐hr dietary recall. The relationship between dietary variables and sleep quality was examined using multivariable logistic regression models. Logistic regression analysis indicated that better diet quality, which features greater food diversity, higher ingestion of fruits and fish, along with higher seafood consumption, lower eggs consumption, and higher total energy intake, was significantly associated with lower risk of poor sleep quality in the crude model and the fully adjusted model with adjustment for gender, age, self‐rated health condition, self‐assessed mental stress, smoking, hypertension, and BMI. Therefore, we reached a conclusion that diet quality and certain food consumption were related to sleep quality. Although the associations observed in the cross‐sectional study require further investigation in prospective studies, dietary intervention, such as enhancement in food diversity and consumption of fruits and seafood, might serve as a probable strategy for sleep improvement.

## 1 INTRODUCTION

Adequate and quality sleep are both crucial to ideal health conditions. The recommended hours of sleep by the National Sleep Foundation are 7–9 hr for adults to maintain health (Hirshkowitz et al., 2015). A growing body of evidence suggests that poor sleep quality elevates the risk of overweight or adiposity (Cappuccio et al., 2008) and other metabolic diseases (Ju & Choi, 2013; Shan et al., 2015). It is especially noteworthy that short sleep duration (generally the amount of time in bed spent asleep less than 7 hr; Dashti et al., 2016) is linked to high levels of inflammation, low levels of cognition, and all‐cause mortality (Campanini et al., 2017; Dashti et al., 2016; Huang, Wahlqvist, & Lee, 2013). A large number of researches imply that the prevalence of poor sleep quality is between 6.6% and 29.4% among Chinese adults, compared with that between 25.6% and 48.1% reported by researches abroad (Del Brutto et al., 2016; Katagiri, Asakura, Kobayashi, Suga, & Sasaki, 2014; Lohsoonthorn et al., 2013). Therefore, the high prevalence and unfavorable outcomes have rendered poor sleep quality, an increasing concern for public health. Apart from genetic factors, a plenty of studies have demonstrated that many other factors are related to sleep, such as gender, age, occupation, psychological state (Haseli‐Mashhadi et al., 2009; Knutson, 2013). Nevertheless, it has been recognized that dietary modification is implicated in sleep alteration. One diet intervention study found that lower fiber and high saturated fat intake were associated with less slow wave sleep in normal adults (St‐Onge, Roberts, & Shechter, 2016). Another randomized clinical trial among obese men with chronic insomnia showed that compared to the controls, the diet group, in which 300–500 kcal/day less energy intake and optimized nutrient composition were recommended, had shorter objective sleep onset latency after intervention (Tan et al., 2016). A cohort study in Rotterdam found that increase in each percent of sleep efficiency was related to reduction of 11.1kcal energy (Dashti et al., 2016). One cross‐sectional study among Japanese workers indicated that there was an association between self‐reported poor sleep quality and higher percentage energy intake for protein (Tanaka et al., 2013). Another three studies, respectively, suggested that poor sleep quality was related to less consumption of vegetables, fish, or both (Del Brutto et al., 2016; Huang et al., 2013; Katagiri et al., 2014). What is more, exploring the correlation of sleep quality and diet is essential because sleep quality cannot be autonomously improved easily while diet quality can (Katagiri et al., 2014).

However, most epidemiology studies with respect to exploring the relationship between sleep quality and diet use only one way of diet assessment. In addition, diet in China is distinct from that in western countries, even other Asian countries. Hence, in the background of Chinese diet our study aimed to determine whether an association between diet and sleep quality existed in Chinese urban adults with evaluating both short‐term and long‐term diet via diet quality, certain food consumption, and dietary nutrients intake.

## 2 MATERIALS AND METHODS

### 2.1 Participants

The data used in the present analysis are from the Chinese Urban Adults Diet and Health Study, a cross‐sectional study. From March to July 2016, a multi‐stage sampling method was used to recruit participants from eight cities locating in China. First, two first‐tier cities (Beijing, Guangzhou), and six second‐tier or third‐tier cities (Jilin, Xuchang, Wuhu, Chenzhou, Lanzhou, and Chengdu) were purposively chosen according to economic status and geographical location. Then, the convenience sampling method was used to select two communities from each first‐tier city and one community from every non‐first‐tier city. On the basis of resident registration, the random sampling was conducted in the communities selected from aforementioned eight cities to recruit potential subjects. Inclusion criteria were as follows: adults between 18 and 75 years old and natives of an urban area or who have lived in urban areas for more than 1 year. A total of 1806 subjects were invited to participant in the telephone survey conducted by local trained medical workers. A total of 1,739 individuals completed the survey after following exclusion criteria had been performed: individuals with disability, pregnant or lactating women, individuals with an infectious disease or mental health or memory problems. Data derived from 1,548 participants remained in the final analysis as 177 participants administered a different version of dietary questionnaire from others, and 14 participants' data were incomplete in terms of education level, household monthly income, self‐rated health condition, self‐assessed mental stress.

Written consent was obtained by local health professionals from all participants prior to the commencement of the study. The Chinese Urban Adults Diet and Health Study was approved by the Medical Ethics Research Board of Peking University (No.IRB00001052‐15059).

### 2.2 Data collection

#### 2.2.1 Sociodemographic and lifestyle factors

Sociodemographic characteristics lifestyle factors including gender, age, ethnicity, education level, marital status, monthly household income, self‐rated health condition, self‐assessed mental stress, and smoking were self‐reported in an interviewer‐administered questionnaire. Metabolic equivalents (METs), categorized into three levels, were used to assess the intensity of physical activities from the International Physical Activity Questionnaire (short version). A pilot survey for questionnaire testing had been done prior to the formal survey.

#### 2.2.2 Anthropometric measurements

Anthropometric measurements including weight, height, and blood pressure were performed during the field work by trained investigators. Standardized procedures and identical equipment were used to obtain weight to the nearest 0.1 kg and height to the nearest 0.1 cm. The average value of two measurements was taken for analysis. Body mass index (BMI) was computed as weight in kg divided by height in meters squared. In our analysis, the criterion for hypertension was an individual with an average systolic BP >140 mmHg and/or an average diastolic BP >90 mmHg or who self‐reported a previous diagnosis of hypertension. Fasting blood samples were drawn in the morning and tested for fasting blood glucose and blood lipid levels. An individual with fasting blood glucose ≥7.0 mmol/L or a previous diagnosis of diabetes was deemed to a diabetic. An individual with TC ≥6.22 mmol/L and/or TG ≥2.26 mmol/L and/or HDL‐D ＜1.04 mmol/L and/or LDL‐D ≥4.14 mmol/L or a previous diagnosis of dyslipidemia was determined dyslipidemia.

#### 2.2.3 Sleep quality

Sleep quality was evaluated by the Chinese version of the Pittsburg Sleep Questionnaire Index (PSQI). The PSQI is composed of 18 self‐assessment items which are directed at assessing sleep quality from seven dimensions, including subjective sleep quality, sleep latency, sleep duration, sleep efficiency, sleep disturbances, use of sleep medication, and daytime dysfunction. The range of total score is from 0 to 21 calculated by summing the score of every dimension (range 0–3). Higher total score denotes poorer sleep quality.

#### 2.2.4 Diet quality

Chinese Healthy Diet Index (CHDI) is established by Chinese Center for Diseases Prevention and Control in 2017. As a kind of diet evaluation index, it aims to evaluate the diet quality and characteristics of Chinese adults, based on 2016 Dietary Guidelines and Food Guide Pagoda for Chinese. According to core items and key recommendations of Dietary Guidelines, CHDI contains 13 components, including nine food groups (refined grains, whole grains, dry bean and tuber, total vegetables, dark green and orange vegetables, fruits, dairy, soybean, meat and eggs, fish, shellfish, and mollusk), food variety, calories from saturated fatty acid, empty calories, and sodium intake. The total score of CHDI, 100 points, is computed by summing the score of each component, with a higher score representing a better diet quality. The maximum score of six items is 10 points separately, with the left components being five points, respectively. The standard for maximum and minimum score of every component is defined based on recommendation intake of food and nutrients in Dietary Guidelines. In our analysis, a single 24‐hr dietary recall underlies the computation of CHDI total score.

#### 2.2.5 Food consumption

The daily consumption of 19 common food categories for analysis was measured using a semiquantitative Food Intake Frequencies Questionnaire (semi‐FFQ): (1) cereals; (2) tubers; (3) fresh vegetables; (4) fruits; (5) poultry and livestock; (6) seafood; (7) freshwater products; (8) eggs; (9) milk and dairy products; (10) soybeans and soybean products; (11) nuts; (12) water; (13) salt; (14) cooking oil; (15) pickled food; (16) sugar‐sweetened beverage; (17) tea; (18) coffee; (19) alcoholic drinks.

#### 2.2.6 Nutrients intake

Intake of daily nutrients and total energy which were collected via a single 24‐hr dietary recall were calculated on the basis of the Chinese Food Composition Table (second edition; Y Y, 2009) coupled with the nutrition information packaging. A validated 24‐hr recall is generally considered sufficient to generalize overall eating patterns at the population level (Grandner, Jackson, Gerstner, & Knutson, 2014). In addition, three noncontinuous 24‐hr dietary recalls were conducted in 205 participants, among which there was no significant difference in total energy.

### 2.3 Statistics analysis

Sleep quality was dichotomized by the total score of PSQI at the cut‐off point of 7 (good, score of 0–6; poor, score of 7 and above; Jin, Zhou, & Peng, 2018). Food group consumption was energy‐adjusted using a density model and presented as grams or milliliters per 1,000 kcal of energy. Nutrients were presented as percentage energy for protein, carbohydrate, and fat, grams or milligrams or microgram per 1,000 kcal of energy for other nutrients. Continuous variables were described as median (25th, 75th) after normality tests and categorical variables were described as percentage. Differences in dietary and confounding variables between sleep groups were analyzed used nonparametric tests for non‐normal continuous variables and binary logistic regression adjusted for gender, age, or both for categorical variables.

In order to further examine the association between sleep quality and dietary factors which showed statistically significance in the results of aforementioned univariate analysis, we used binary logistic regression with sleep quality as the dependent variable. Separate regression models were applied to each dietary variables selected. Continuous variables of dietary factors were divided into quartiles (Q1 was the lowest and the reference and Q4 was the highest). Testing for linear trend was performed by applying the median of every dietary variable to each category and analyzing them as continuous variables in logistic regression. Since only a minority reported they had ever consumed tea, coffee, beverage, and alcohol, consumption of them was described as dichotomous variables based on whether participants consumed at least once per month (Yes) or never (No). Three models were evaluated. First, unadjusted associations were examined. Then, these associations were adjusted for gender and age. Finally, adjustment was further made for gender, age, self‐rated health condition, self‐assessed mental stress, smoking, hypertension, BMI, selected from the results of aforementioned analysis, in the third model. All statistical procedures were performed using SAS 9.4 (SAS Institute). *p*‐Values were two‐tailed, and *p* < 0.05 was considered statistically significant.

## 3 RESULTS

Sociodemographic factors, lifestyle factors, and health‐related indicators of the participants included in this analysis are shown between two groups in Table 1. Overall, 25.2% (390) of the total had a poor sleep quality. Women, individuals above 45‐year‐old, or with normal BMI and above, current‐smokers tended to have a higher risk of poor sleep quality. Besides, poor sleep quality was significantly associated with hypertension, sub‐health, or illness of self‐assessment compared with health. There was also a positive association between the prevalence of poor sleep quality and the frequency of self‐rated mental stress. No significant association was seen between sleep quality and other characteristics.

**Table 1:** Basic characteristics of Chinese urban adults by sleep quality (*N* %)

	Sleep quality		OR（95% CI)^a^
	Good	Poor	
	*N* = 1,158	*N* = 390	
Sociodemographic factors			
Gender			
Male	436 (37.7)	116 (29.7)	Ref
Female	722 (62.3)	274 (70.3)	1.04 (0.84–1.30)
Age (y)			
18–30	228 (19.7)	38 (9.7)	Ref
30–45	219 (18.9)	42 (10.8)	1.56 (1.04–2.34)
45–65	366 (31.6)	157 (40.3)	3.47 (2.46–4.92)
≥65	345 (29.8)	153 (39.2)	4.05 (2.85–5.75)
Cities			
First‐tier cities	374 (32.3)	126 (32.3)	Ref
Second‐tier cities	267 (23.1)	75 (19.2)	0.89 (0.64–1.24)
Third‐tier cities	517 (44.6)	189 (48.5)	1.19 (0.91–1.56)
Minority			
Han Nationality	1,131 (97.7)	380 (97.4)	Ref 1.11 (0.52–2.35)
Hon‐Han Nationality	27 (2.3)	10 (2.6)	
Occupational status^b^			
Employed	478 (41.3)	114 (29.2)	Ref
Unemployed	57 (4.9)	20 (5.1)	1.19 (0.68–2.09)
Retired	440 (38)	195 (50)	1.12 (0.79–1.58)
Never work	183 (15.8)	60 (15.4)	1.17 (0.79–1.73)
Education level			
Junior high school or below	376 (32.5)	176 (45.1)	Ref
Senior high school	445 (38.4)	138 (35.4)	0.81 (0.61–1.06)
Bachelor's degree or above	337 (29.1)	76 (19.5)	0.79 (0.55–1.12)
Marital status^c^			
Unmarried	193 (16.7)	32 (8.2)	Ref
Married	891 (76.9)	316 (81)	1.10 (0.50–2.46)
Divorced	71 (6.1)	41 (10.5)	1.36 (0.56–3.35)
Household monthly income (rmb: yuan)			
<5,000	561 (48.4)	206 (52.8)	Ref
5,000–9999	361 (31.2)	116 (29.7)	0.91 (0.70–1.20)
>10,000	236 (20.4)	68 (17.4)	0.80 (0.58–1.10)
Lifestyle factors			
Self‐rated health condition			
Healthy	485 (41.9)	91 (23.3)	Ref
Sub‐healthy	523 (45.2)	201 (51.5)	2.07 (1.56–2.74)
Diseased	98 (8.5)	81 (20.8)	3.41 (2.33–5.00)
Recovering of diseases	22 (1.9)	9 (2.3)	2.03 (0.90–4.59)
Unknown	30 (2.6)	8 (2.1)	1.58 (0.69–3.61)
Self‐rated mental stress			
Always	15 (1.3)	22 (5.6)	Ref
Often	257 (22.2)	99 (25.4)	0.29 (0.14–0.61)
Sometimes	520 (44.9)	154 (39.5)	0.21 (0.10–0.42)
Seldom	351 (30.3)	110 (28.2)	0.15 (0.07–0.31)
Unknown	15 (1.3)	5 (1.3)	0.21 (0.06–0.73)
Intensity of physical activities			
High	280 (24.2)	99 (25.4)	Ref
Medium	608 (52.5)	194 (49.7)	0.88 (0.66–1.18)
Low	270 (23.3)	97 (24.9)	1.07 (0.77–1.50)
Smoking			
Never smoke	858 (74.1)	296 (75.9)	Ref
Previous smoker	139 (12)	34 (8.7)	0.94 (0.57–1.53)
Current smoker	152 (13.1)	59 (15.1)	1.61 (1.05–2.46)
Unknown	9 (0.8)	1 (0.3)	0.38 (0.05–3.02)
Health‐related indicators			
Dyslipidemia			
Yes	343 (29.6)	150 (38.5)	Ref 0.83 (0.64–1.06)
No	815 (70.4)	240 (61.5)	
Hypertension			
Yes	386 (33.3)	179 (45.9)	Ref 0.75 (0.58–0.98)
No	772 (66.7)	211 (54.1)	
Diabetes			
Yes	143 (12.3)	60 (15.4)	Ref 0.96 (0.69–1.35)
No	1,015 (87.7)	330 (84.6)	
BMI			
Underweight	73 (6.3)	9 (2.3)	Ref
Normal	569 (49.1)	188 (48.2)	2.16 (1.04–4.46)
Overweight or obese	516 (44.6)	193 (49.5)	2.15 (1.03–4.48)

Abbreviation: Ref, reference; OR, odds ratios; CI, confidence interval.

a All the sociodemographic factors, lifestyle factors, and health‐related indicators were presented as N (%), and differences between groups were compared using binary logistics regression adjusted for gender, age, or both.

b One missing value in the poor sleep quality group.

c Three missing values in the good sleep quality group and one in the poor sleep quality group.

### 3.1 Diet quality

Total and individual score of CHDI between two sleep quality groups are shown in Table 2. The total score of CHDI was found significantly higher in the group of good sleep quality. Moreover, the components of CHDI which showed significant correlations with sleep quality were food variety, fruits and fish, shellfish, and mollusk.

**Table 2:** Total and individual CHDI score by sleep quality (Median (P25, P75))

	Sleep quality		*p* a
	Good	Poor	
	*N* = 1,158	*N* = 390	
CHDI(score)	56.8 (48.1, 64.7)	54.2 (46.5, 62.6)	0.002
CHDI items(score)			
Food variety	7.1 (4.3, 10.0)	5.7 (2.9, 10.0)	0.004
Refined grains	5.0 (5.0, 5.0)	5.0 (5.0, 5.0)	0.141
Whole grain, dry bean, and tuber	1.8 (0.0, 5.0)	2.4 (0.0, 5.0)	0.213
Total vegetables	3.5 (2.0, 5.0)	3.8 (1.9, 5.0)	0.380
Dark green and orange vegetables	1.5 (0.0, 4.1)	1.1 (0.0, 3.9)	0.226
Fruit	5.1 (0.0, 10.0)	3.1 (0.0, 10.0)	0.006
Dairy	0.0 (0.0, 0.7)	0.0 (0.0, 0.8)	0.317
Soybean	0.0 (0.0, 10.0)	0.0 (0.0, 10.0)	0.330
Meat and egg	5.0 (3.4, 5.0)	5.0 (3.2, 5.0)	0.757
Fish, shellfish, and mollusk	0.0 (0.0, 2.3)	0.0 (0.0, 0.0)	0.018
Calories from SFA	10.0 (10.0, 10.0)	10.0 (10.0, 10.0)	1.000
Sodium	4.7 (0.9, 7.2)	4.7 (1.0, 7.4)	0.854
Empty calories	10.0 (9.1, 10.0)	10.0 (9.0, 10.0)	0.463

Abbreviations: SFA, saturated fatty acid.

a The total and individual CDHI cores were presented as median (P25‐P75), and differences between groups were compared using nonparametric test.

In multivariable logistic regression analysis of CHDI and sleep quality, a higher total score of CHDI was inversely related to poor sleep quality in the crude model. After adjustment for gender and age, this correlation still remained, and it was the same case with fully adjusted model (Table 5). The associations between the individual score of 3 CHDI components and sleep quality were likewise statistically significant in fully adjusted model, which denoted greater food varieties, higher consumption of fruits, fish, shellfish, and mollusk were correlated with lower risk of poor sleep quality.

### 3.2 Food groups

Table 3 presents the distribution of food consumption between two sleep quality groups. Consumption of fresh vegetables, seafood, eggs, and beverage differentiated significantly between two groups. Poor sleep quality group reported lower intake of seafood and beverage, contrary to higher intake of fresh vegetables and eggs.

**Table 3:** Consumption of food by sleep quality (Median (P25, P75))

	Sleep quality		*p* a
	Good	Poor	
	*N* = 1,158	*N* = 390	
Cereals(g/1,000 kcal)	162.8 (96.9, 234.2)	170.5 (102.7, 264.0)	0.112
Tubers(g/1,000 kcal)	10.3 (3.0, 26.0)	10.6 (3.3, 27.6)	0.634
Fresh vegetables(g/1,000 kcal)	151.4 (73.2, 271.2)	188.3 (87.1, 314.0)	0.002
Fruits(g/1,000 kcal)	68.2 (29.4, 139.0)	76.0 (28.0, 147.3)	0.639
Poultry and livestock (g/1,000 kcal)	35.6 (14.3, 68.3)	34.5 (13.0, 71.2)	0.650
Seafood(g/1,000 kcal)	1.3 (0.0, 8.9)	0.2 (0.0, 5.8)	0.006
Freshwater products (g/1,000 kcal)	4.8 (0.3, 14.8)	4.3 (0.0, 12.0)	0.235
Eggs(g/1,000 kcal)	23.8 (10.3, 40.5)	27.7 (14.3, 45.4)	0.005
Milk and dairy products (g/1,000 kcal)	36.9 (0.0, 114.8)	44.2 (0.0, 125.2)	0.427
Soybeans and soybean products (g/1,000 kcal)	13.5 (3.8, 33.4)	14.3 (4.2, 37.6)	0.415
Nuts(g/1,000 kcal)	3.6 (0.0, 13.3)	3.3 (0.0, 13.4)	0.464
Water(ml/1,000 kcal)	800.0 (375.0, 1525.0)	775.0 (275.0, 1,500.0)	0.322
Salt(g/1,000 kcal)	5.8 (3.1, 9.3)	5.7 (3.2, 10.0)	0.600
Cooking oil(g/1,000 kcal)	14.5 (7.1, 24.0)	14.9 (7.1, 27.1)	0.503
Pickled food(g/1,000 kcal)	0.6 (0.0, 3.9)	0.7 (0.0, 4.5)	0.469
Sugar‐sweetened beverage (ml/1,000 kcal)	0.0 (0.0, 0.0)	0.0 (0.0, 0.0)	0.044
Tea(ml/1,000 kcal)	4.3 (0.0, 133.0)	0.0 (0.0, 175.5)	0.422
Coffee(ml/1,000 kcal)	0.0 (0.0, 0.0)	0.0 (0.0, 0.0)	0.168
Alcoholic drinks(ml/1,000 kcal)	0.0 (0.0, 3.8)	0.0 (0.0, 1.1)	0.225

a The consumption of food groups was presented as median (P25‐P75), and differences between groups were compared using nonparametric test.

The results of multivariable logistic regression analysis of food consumption and sleep quality are indicated in Table 5 as well. Lower seafood consumption and higher eggs consumption significantly increased the risk of poor sleep quality in both unadjusted and adjusted analyses. Intake of fresh vegetables was positively associated with the risk of poor sleep quality in the unadjusted model, while this association did not exist in model 2 and model 3 after adjustment for potential confounders. Participants who reported to have consumed beverage had an elevated risk of poor sleep quality in the crude model, whereas this correlation did not meet the threshold for statistical significance in both partially adjusted and fully adjusted models.

### 3.3 Nutrients

Distribution of nutrients intake between two sleep quality groups is shown in Table 4. Total energy intake was found lower in poor sleep quality group，apart from which no association of other nutrients with sleep quality was observed in univariate analysis. In the following multivariable logistic regression, a positive correlation was identified between total energy intake and the risk of poor sleep quality in both unadjusted and fully adjusted models (Table 5).

**Table 4:** Intake of nutrients by sleep quality (Median P25, P75)

	Sleep quality		*p* a
	Good	Poor	
	*N* = 1,158	*N* = 390	
Energy (kJ)	6,726.46 (5,045.55, 8,950.48)	6,178.39 (4,706.71, 8,271.08)	0.002
Protein (%E)	0.14 (0.12, 0.17)	0.14 (0.12, 0.17)	0.353
Fat (%E)	0.34 (0.26, 0.42)	0.35 (0.25, 0.43)	0.885
Carbohydrate (%E)	0.58 (0.48, 0.66)	0.57 (0.47, 0.67)	0.824
Diet fiber (g/1,000 kcal)	5.41 (3.80, 7.90)	5.46 (3.85, 7.72)	0.660
Cholesterol (g/1,000 kcal)	169.29 (54.37, 285.61)	156.08 (45.33, 303.34)	0.784
Vitamin A (μg/1,000 kcal)	196.28 (116.84, 327.8)	202.09 (112.81, 307.85)	0.577
Retinol (μg/1,000 kcal)	69.32 (23.17, 123.44)	71.6 (17.01, 130.3)	0.920
Thiamin (mg/1,000 kcal)	0.48 (0.39, 0.59)	0.49 (0.38, 0.61)	0.401
Riboflavin (mg/1,000 kcal)	0.44 (0.34, 0.59)	0.45 (0.33, 0.63)	0.402
Niacin (mg/1,000 kcal)	6.64 (5.43, 8.46)	6.5 (5.38, 8.3)	0.495
Vitamin C (mg/1,000 kcal)	35.59 (18.99, 60.91)	35.18 (17.05, 60.73)	0.590
Vitamin E (mg/1,000 kcal)	13.02 (8.43, 19.41)	13.38 (8.74, 19.95)	0.249
α‐Vitamin E (mg/1,000 kcal)	3.83 (2.58, 5.49)	3.99 (2.69, 5.5)	0.363
Folic acid (μg/1,000 kcal)	109.34 (77.44, 163.93)	110.63 (76.66, 169.67)	0.949
Vitamin B_6_ (mg/1,000 kcal)	0.53 (0.41, 0.66)	0.52 (0.4, 0.63)	0.167
Vitamin B_12_ (μg/1,000 kcal)	1.11 (0.45, 2.21)	1.07 (0.38, 1.95)	0.181
Vitamin D (μg/1,000 kcal)	0.35 (0.05, 2.33)	0.31 (0.06, 1.95)	0.489
Ca (mg/1,000 kcal)	208.28 (138.82, 313.59)	204.07 (124.73, 334.49)	0.672
P (mg/1,000 kcal)	512.18 (436.21, 581.78)	513.06 (435.73, 591.89)	0.371
K (mg/1,000 kcal)	906.93 (711.22, 1,142.26)	901 (718.19, 1,140.98)	0.905
Na (mg/1,000 kcal)	2,590.3 (1837.43, 3,718.88)	2,597.3 (1779.5, 3,701.27)	0.807
Mg (mg/1,000 kcal)	144.23 (118.92, 175.3)	143.68 (120.85, 178.45)	0.596
Fe (mg/1,000 kcal)	10.25 (8.77, 12.09)	10.11 (8.73, 12.16)	0.861
Zn (mg/1,000 kcal)	5.38 (4.77, 6.2)	5.42 (4.67, 6.46)	0.620
Se (μg/1,000 kcal)	20.57 (15.89, 27.15)	20.84 (15.19, 27.3)	0.760
Cu (mg/1,000 kcal)	0.94 (0.77, 1.17)	0.94 (0.78, 1.18)	0.556
Mn (mg/1,000 kcal)	2.81 (2.28, 3.45)	2.81 (2.29, 3.52)	0.653

a The intake of nutrients was presented as median (P25, P75), and differences between groups were compared using nonparametric test.

**Table 5:** Unadjusted and adjusted odds ratios and 95% confidence intervals for poor sleep quality among 1,548 Chinese urban adults

	Model	Q1 (Lowest)	Q2	Q3	Q4 (Highest)	*p* for trend^a^
		*N* = 387	*N* = 387	*N* = 387	*N* = 387	
CHDI(score)	Model1^b^	Ref	1.01 (0.74, 1.38)	0.78 (0.56, 1.07)	0.60 (0.43, 0.84)*	0.003
	Model2^c^	Ref	0.97 (0.70, 1.33)	0.76 (0.55, 1.06)	0.58 (0.41, 0.82)*	0.006
	Model3^d^	Ref	0.95 (0.68, 1.33)	0.80 (0.57, 1.12)	0.62 (0.43, 0.88)*	0.031
CHDI items						
Food variety	Model1^b^	Ref	0.82 (0.60, 1.13)	0.81 (0.57, 1.14)	0.63 (0.46, 0.85) *	0.004
	Model2^c^	Ref	0.81 (0.58, 1.12)	0.82 (0.58, 1.17)	0.65 (0.47, 0.89) *	0.010
	Model3^d^	Ref	0.83 (0.59, 1.17)	0.85 (0.59, 1.22)	0.70 (0.50, 0.97) *	0.043
Fruit	Model1^b^	Ref	0.78 (0.54, 1.13)	0.65 (0.47, 0.90)*	0.71 (0.53, 0.94) *	0.006
	Model2^c^	Ref	0.70 (0.48, 1.02)	0.58 (0.42, 0.81)*	0.65 (0.48, 0.87) *	0.001
	Model3^d^	Ref	0.69 (0.46, 1.01)	0.60 (0.42, 0.84)*	0.71 (0.52, 0.97) *	0.012
Fish, shellfish, and mollusk	Model1^b^	Ref	0.91 (0.47, 1.77)	0.70 (0.53, 0.92) *		0.012
	Model2^c^	Ref	1.00 (0.51, 1.98)	0.71 (0.53, 0.95) *		0.019
	Model3^d^	Ref	1.01 (0.50, 2.03)	0.73 (0.54, 0.98) *		0.036
Total energy(kJ)	Model1^b^	Ref	0.86 (0.63, 1.17)	0.75 (0.54, 1.03)	0.61 (0.44, 0.84)*	0.002
	Model2^c^	Ref	0.87 (0.63, 1.19)	0.77 (0.56, 1.07)	0.73 (0.52, 1.04)	0.074
	Model3^d^	Ref	0.85 (0.61, 1.19)	0.79 (0.56, 1.11)	0.70 (0.49, 1.00)*	0.055
Food categories						
Fresh vegetables (g/1,000 kcal)	Model1^b^	Ref	0.78 (0.55, 1.10)	1.38 (1.00, 1.91)*	1.42 (1.03, 1.95)^*^	0.002
	Model2^c^	Ref	0.66 (0.46, 0.95)*	1.10 (0.79, 1.55)	1.03 (0.73, 1.44)	0.243
	Model3^d^	Ref	0.71 (0.49, 1.03)	1.12 (0.79, 1.59)	1.11 (0.78, 1.57)	0.149
Seafood (g/1,000 kcal)	Model1^b^	Ref	0.69 (0.39, 1.22)	0.89 (0.67, 1.18)	0.67 (0.50, 0.90)*	0.014
	Model2^c^	Ref	0.76 (0.42, 1.37)	0.96 (0.72, 1.29)	0.68 (0.50, 0.91)*	0.012
	Model3^d^	Ref	0.73 (0.40, 1.34)	1.02 (0.75, 1.37)	0.68 (0.49, 0.93)*	0.015
Eggs (g/1,000 kcal)	Model1^b^	Ref	1.27 (0.90, 1.77)	1.25 (0.89, 1.75)	1.70 (1.23, 2.36)*	0.002
	Model2^c^	Ref	1.16 (0.82, 1.64)	1.10 (0.78, 1.55)	1.45 (1.04, 2.04)*	0.033
	Model3^d^	Ref	1.22 (0.85, 1.74)	1.17 (0.82, 1.68)	1.55 (1.09, 2.20)*	0.020
		No	Yes			
Beverage (ml/1,000 kcal)	Model1^b^	Ref	0.74 (0.57, 0.97)*			—
	Model2^c^	Ref	1.22 (0.90, 1.65)			—
	Model3^d^	Ref	1.20 (0.87, 1.64)			—

Abbreviations: Q, quartile; Ref, reference.

a The results of multivariable binary logistic regression were showed as unadjusted and adjusted odds ratios and 95% confidence intervals for poor sleep quality. Testing for linear trend was performed by applying the median of every dietary variable to each category and analyzing them as continuous variables in logistic regression.* indicated *p* < 0.05.

b Model1 was unadjusted.

c Model2 was adjusted for gender and age.

d Model3 was adjusted for gender, age, self‐rated health condition, self‐assessed mental stress, smoking, hypertension, BMI.

## 4 DISCUSSION

The present study examined the associations between dietary factors and sleep quality. Our results suggest that better sleep quality is significantly related to better diet quality, which features greater food varieties, higher consumption of fruits and fish, especially seafood, along with lower ingestion of eggs and higher total energy intake. Furthermore, all of the abovementioned associations were dose‐dependent and still exhibited statistical significance after adjustment for a series of potential confounding variables.

Prior studies mainly focused on individual nutrients and foods, so they may not explore their synergistic effects. In addition, as different countries have different dietary patterns and dietary intake, it is reasonable to examine the diet holistically on a basis of dietary patterns or diet quality, which have been suggested to better reflect the complexity of dietary intake (Hu, 2002). Further, studies focusing on food groups and overall diet are also necessary, as they would provide an approachable benchmark for alternations in food choice and lifestyle (Katagiri et al., 2014).

The current study provided new evidence of a positive association between diet quality, food diversity, and sleep quality; however, we only found a similar result in one study (van Lee et al., 2017). This study came to a conclusion that good sleep quality was associated with a better diet quality, which is consistent with our study. Although it was derived from a cohort of healthy pregnant women instead of general population in Singapore, this result was still comparable to ours. It is similar to ours that sleep quality was also assessed by PSQI, and diet quality was also evaluated by a kind of diet index—Healthy Eating Index in this cohort study. Among the findings, our study suggested that the correlation of diet with sleep quality overlaps that of sleep efficiency or sleep duration in other studies, both of which belong to the seven components of sleep quality measured by PSQI. Short sleep duration (generally the amount of time in bed spent asleep <7 hr; Dashti et al., 2016) and low sleep efficiency (generally the percentage of sleep duration in bed <85%; St‐Onge, Mikic, & Pietrolungo, 2016) commonly denote poor sleep quality. Compared to adequate sleep, the relationship between short sleep duration and poor diet quality as assessed with various diet quality scores has been extensively shown in Iranian students (Haghighatdoost, Karimi, Esmaillzadeh, & Azadbakht, 2012), European adolescents (Bel et al., 2013), and postmenopausal women (Stern et al., 2014). In a cohort‐based study, healthier diet quality assessed by Alternative Healthy Eating Index diet quality (AHEI) was associated with both an increase in sleep duration and sleep efficiency (Mossavar‐Rahmani et al., 2017). Additionally, short sleepers were also reported to tend to have lower food diversity evaluated by Dietary Diversity Score (Haghighatdoost et al., 2012).

With regard to the score of individual item in CHDI, we noticed a significant correlation of a higher score of fruit consumption with lower risk of poor sleep quality, which is supported by a study indicative of increased whole fruit intake as one component of AHEI associated with higher sleep efficiency (Mossavar‐Rahmani et al., 2017). Fruits consumption was likewise found to be positively related to sleep duration (Gong et al., 2017; Haghighatdoost et al., 2012). The findings that higher adherence to Mediterranean diet (Campanini et al., 2017) or Chinese modern dietary patterns (Yu et al., 2017), both of which are characterized by high intake of fruits and other specific foods, were associated with better sleep quality strengthen our result. Furthermore, a review implied fruits may promote sleep duration and efficiency (Peuhkuri, Sihvola, & Korpela, 2012). The mechanisms underlying the association of fruits with sleep quality may be due to melatonin or serotonin, and antioxidant capacity of fruits. Previous studies have reported a correlation between disordered sleep and oxidative stress (Tsaluchidu, Cocchi, & Tonello, 2008), and it has been established that melatonin, the sleep‐promoting neurosecretory hormone, and the neurotransmitter serotonin, an intermediary product of melatonin, regulate sleep cycle (Peuhkuri et al., 2012). Therefore, fresh fruits with high dietary concentration of melatonin or serotonin and antioxidant substance, such as kiwifruits (Lin, Tsai, & Fang, 2011), tart cherries (St‐Onge, Mikic, et al., 2016), may be beneficial in improving sleep quality.

There have been other studies revealing an inverse association between the PSQI score and oily fish among adults living in rural coastal Ecuador (Del Brutto et al., 2016) and Japanese female workers (Katagiri et al., 2014). A cross‐sectional study among Japanese adults reported that intake of fish and shellfish was likewise correlated with sleep duration in male (Komada et al., 2017). In addition, another cohort study found that higher compliance with Mediterranean diet, which also features moderate, or even high, consumption of fish, olive oil, fruits, and vegetables, was associated with better sleep quality (Campanini et al., 2017). Our results are consistent with these studies. The results derived from both daily intake of seafood based on the semi‐FFQ and individual score of fish from CHDI calculated by the 24‐hr dietary recall suggested a significant association between fish, especially seafood, and sleep quality in the present study. Several factors could account for this association. First, in animal foods, melatonin concentrations were found higher in fish and eggs than those in meat (Meng et al., 2017). Moreover, seafood, especially fatty fish, is good resources of long‐chain omega‐3 polyunsaturated fatty acids and vitamin D, both of which have been reported to be positively correlated with sleep quality (Del Brutto et al., 2016; Hansen et al., 2014). Omega‐3 fatty acids play an important role in the secretion of serotonin, which is a biogenic amine involved in sleep regulation (Monti, 2011). Vitamin D is implicated in sleep–wake cycle (Gominak & Stumpf, 2012).

Interestingly, our study was suggestive that eggs intake appeared to be conversely with sleep quality. So far, there is a lack of researches regarding the correlation of eggs ingestion and sleep quality. Only two cross‐sectional studies conducted in Japan and China, respectively, reported that individuals with higher compliance with certain dietary pattern which features high consumption of eggs and other foods were less likely to have insomnia symptoms (Kurotani et al., 2015; Yu et al., 2017). Possible explanations for the effect of eggs on sleep may be related to cholesterol. Although eggs are rich in melatonin as mentioned before, eggs are the most principal source of cholesterol for Chinese in recent decade (Su, Jia, Wang, Wang, & Zhang, 2015) and we also found higher egg consumption contributed to more cholesterol intake significantly in our study population (Spearman correlation coefficient = 0.283, *p* < 0.001). Furthermore, a negative correlation was found between dietary cholesterol and sleep duration in men (Santana et al., 2012), although this correlation had not been captured in our study. Therefore, further studies on the associations between eggs and sleep quality and underlying mechanisms are needed.

There has been emerging information surrounding higher sugar‐sweetened beverage consumption related to short sleep duration (Katagiri et al., 2014; Kleiser et al., 2017). However, in the current study the negative correlation of sugar‐sweetened beverage consumption with sleep quality did not exhibit significance after considering gender, age, and other confounders. The reason for this phenomenon may attribute to an age‐effect that beverages are generally targeted to young adult consumers, as consumers of sugar‐sweetened beverages were significantly younger than nonconsumers among the participants for analysis( median age 34.1 years vs. 61.8 years, *p* < 0.01). Moreover, our results showed that the middle‐aged and the elder were more likely to be poor sleep quality.

As to the association between total energy intake and sleep quality, our finding is in conflict with most existing studies implying that more energy intake was related to shorter sleep duration and deteriorated sleep parameters such as sleep onset latency (Haghighatdoost et al., 2012; Stern et al., 2014; Tan et al., 2016), yet one study in India reported that insomniacs without co‐morbid compared to normal sleepers consumed significantly less energy (Zadeh & Begum, 2011). Hence, longitudinal studies are imperative to elucidate this discrepancy which remains unclear.

Cow's milk (Gong et al., 2017) and coffee (Kleiser et al., 2017) have been traditionally considered as sleep‐promoting and sleep‐inhibiting beverage, separately, and have been found to be correlated with sleep duration. The lack of signals from milk and dairy products or coffee on sleep quality in our study is noteworthy, but it has to be interpreted with caution due to the large number of nonconsumers among Chinese residents, particularly milk and dairy products consumption (median absolute consumption 71.4 g) far below the recommended daily intake of 300 g.

The current study is on the basis of a cross‐sectional design and is subject to several limitations. First, we observed a couple of dietary factors associated with sleep quality, whereas the inherent limitation of a cross‐sectional study did not allow for causality inference. Second, PSQI is recognized as the most common subjective method to assess sleep quality in large population‐based epidemiology studies all around the world. However, given bias and measurement accuracy, objective methods are warranted in the following researches. Third, regarding a few discrepancies between the present study and others, there still may be residual confounding variables we did not include in the finally adjusted model resulting from either sleep‐influencing ingredients in healthcare products that we failed to identify or the impact of drugs for other chronic conditions on sleeping.

## 5 CONCLUSIONS

In conclusion, better diet quality, particularly higher food diversity, higher consumption of fruits and seafood and lower consumption of eggs were found to be related to better sleep quality in Chinese adults. Although the associations observed in this cross‐sectional study require further investigation in prospective studies, dietary intervention, such as enhancement in food diversity, increment in consumption of fruits and seafood, might serve as a probable strategy for sleep improvement.

## CONFLICT OF INTEREST

All the authors declare no conflict of interest. Transparency Declaration: The lead author affirms that this manuscript is an honest, accurate, and transparent account of the study being reported. The reporting of this work is compliant with STROBE guidelines. The lead author affirms that no important aspects of the study have been omitted and that any discrepancies from the study as planned have been explained.

## ETHICAL STATEMENT

The study conforms to the Declaration of Helsinki, US and does not embrace any human or animal testing. The study's protocols and procedures were ethically reviewed and approved by the Medical Ethics Research Board of Peking University (No. IRB00001052‐15059).
